# Supplementary material for: The Development and Evaluation of a Novel Highly Selective PET Radiotracer for Targeting BET BD1
Source: Pharmaceuticals (Basel). 2024 Sep 27;17(10):1289. doi: 10.3390/ph17101289 (PMC11509907; doi:10.3390/ph17101289)

The Development and Evaluation of a Novel Highly Selective PET Radiotracer for Targeting BET BD1

Yanli Wang, Yongle Wang, Yulong Xu, Leyi Kang, Darcy Tocci and Changning Wang *

Athinoula A. Martinos Center for Biomedical Imaging, Department of Radiology, Massachusetts General Hospital, Harvard Medical School, Charlestown, MA 02129, USA; ywang@mgh.harvard.edu (Y.W.); ywang151@mgh.harvard.edu (Y.W.); yulong.xu@mgh.harvard.edu (Y.X.); lkang5@mgh.harvard.edu (L.K.); darcytocci@brandeis.edu (D.T.)
* Correspondence: Correspondence: cwang15@mgh.harvard.edu

Supplementary Material

A. General information 1

B. Purity analysis 2

C. Chemistry Methods 3-7

A. General information

We preformed study followed by our previous method. We obtained $[^{11}C]CO_2$ via the 14N (p, α)^{11}C reaction with 2.5% oxygen and 11 MeV protons (Siemens Eclipse cyclotron) in nitrogen, and captured on molecular sieves by TRACERlab, FX-MeI synthesizer (General Electric). We obtained [11C]CH4 by reducing $[^{11}C]CO_2$ in the presence of Ni/hydrogen at 350 °C and recirculated it through an oven equipped with I^2, and produced $[^{11}C]CH_3I$ by radical reaction.

For this study, a total of eight male C57BL6 mice, aged 5 months, were employed. All mice research were carried out at Massachusetts General Hospital (PHS Assurance of Compliance No. A3596–01). The Subcommittee on Research Animal Care (SRAC) serves as the Institutional Animal Care and Use Committee (IACUC) for the Massachusetts General Hospital (MGH). To minimize mice (C57BL/6) discomfort, we performed PET/CT imaging in anesthetized (isoflurane) mice. Throughout the process, animal safety is monitored by trained animal technicians. Daily care is the responsibility of the veterinarian. Keep all mice socially in cages, and give them unlimited food and water, and provide extra nutritional supplements as required by the attending veterinarian.

B. Purity analysis

```
Data File C:\Users\P...s\ChemStation\1\Data\XYL\2024-02-2314-53-02YL-CPD31-02232024-PURE.D
Sample Name: YL-CPD31-02232024-PURE
    =====================================================================
    Acq. Operator   : SYSTEM
    Sample Operator : SYSTEM
    Acq. Instrument : HPLC 1 - Analytical              Location :   81
    Injection Date  : 2/23/2024 2:57:40 PM                  Inj :    1
                                                 Inj Volume : 10.000 µl
    Different Inj Volume from Sample Entry!  Actual Inj Volume : 100.000 µl
    Method          : C:\Users\Public\Documents\ChemStation\1\Methods\TEST.M
    Last changed    : 4/5/2021 2:37:34 PM by SYSTEM
    =====================================================================
```

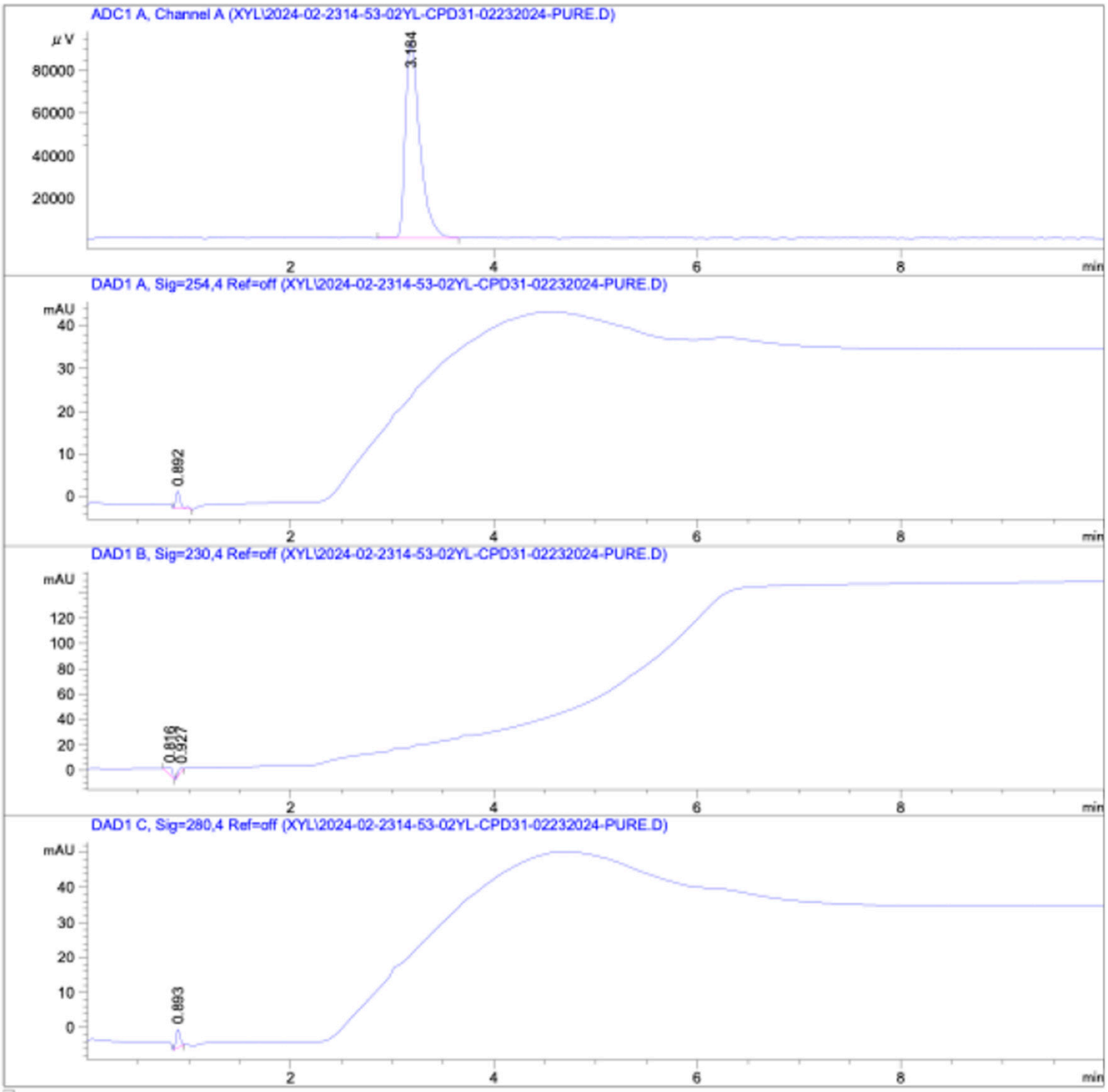

Figure S1. The HPLC chromatogram of [^{11}C]GSK023. Analytic HPLC condition: Agilent Eclipse plus C18, 3.5 µm, 4.6×100 mm, flow rate = 1.0 mL/min, mobile phase = 0.1% formic acid in water / 0.1% formic acid in acetonitrile, gradient method.

C. Chemistry Methods

Materials and Methods: All required reagents and solvents were sourced from established commercial vendors, including Sigma-Aldrich (St. Louis, MO) and Acros Organics. Analytical separations were performed using an Agilent 1100 series HPLC system equipped with a diode-array detector, quaternary pump, vacuum degasser, and autosampler. Thin-layer chromatography (TLC) analyses were conducted on silica gel GF254 plates. NMR spectra were obtained at room temperature using a JEOL NMRECZ500R Spectrometer, operating at frequencies of 500 MHz for ^1H, 471 MHz for ^19F, and 126 MHz for ^13C. Chemical shifts were reported in δ (ppm) with tetramethylsilane (TMS) as the internal standard. Mass spectrometry data were acquired using an Agilent 6310 ion trap mass spectrometer with an electrospray ionization (ESI) source, connected to an Agilent 1200 series HPLC system.

Chemical Analysis: The purity of synthesized compounds was verified using an analytical HPLC method. All compounds were confirmed to have a purity of over 95%.

Synthesis of GSK023 pre and GSK023

(a) To a solution of tert-butyl (3S)-3-(aminomethyl)piperidine-1-carboxylate (5 g, 23.33 mmol, 1 eq) and 1-fluoro-2-nitro-benzene (4.94 g, 35.00 mmol, 3.70 mL, 1.5 eq) in ACM (57 mL) was added K_2CO_3 (16.12 g, 116.66 mmol, 5 eq). The mixture was stirred at 80 °C for 5 hr. TLC indicated tert-butyl (3S)-3-(aminomethyl)piperidine-1-carboxylate was consumed completely and one new spot formed. The reaction mixture was diluted with H_2O (30 mL) and extracted with EtOAc (10 mL * 3). The combined organic layers were washed with brine (5 mL * 3), dried over Na_2SO_4, filtered and concentrated under reduced pressure to give a residue. The residue was purified by column chromatography (SiO_2, Petroleum ether/Ethyl acetate=10/1 to 5/1). Compound tert-butyl (3S)-3-[(2-nitroanilino)methyl]piperidine-1-carboxylate (7 g, 20.66 mmol, 88.56% yield, 99% purity) was obtained as a yellow solid. $(M+H)^+$: 336.2.

(b) To a solution of tert-butyl (3S)-3-[(2-nitroanilino)methyl]piperidine-1-carboxylate (1.5 g, 4.47 mmol, 1 eq) and 5-methyl-6-oxo-1H-pyridine-3-carbaldehyde (797.31 mg, 5.81 mmol, 1.3 eq) in EtOH (20 mL) and H_2O (10 mL) was added $Na_2S_2O_4$ (2.34 g, 13.42 mmol, 2.92 mL, 3 eq) . The mixture was stirred at 120 °C for 3 hr. LC-MS showed tert-butyl (3S)-3-[(2-nitroanilino)methyl]piperidine-1-carboxylate was consumed completely and one main peak with desired m/z was detected. The reaction mixture was diluted with H_2O 50mL and extracted with EtOAc (30mL * 3). The combined organic layers were washed with brine (10 mL * 3), dried over Na_2SO_4, filtered and concentrated under reduced pressure to give a residue. The crude product was triturated with Petroleum ether/Ethyl acetate=3/1 (20ml) at 25 °C for 10 min. Compound tert-butyl (3S)-3-[[2-(5-methyl-6-oxo-1H-pyridin-3-yl)benzimidazol-1-yl]methyl]piperidine-1-carboxylate (900 mg, 2.13 mmol, 47.63% yield) was obtained as a white solid. $(M+H)^+$: 423.2.

(c) To a solution of tert-butyl (3S)-3-[[2-(5-methyl-6-oxo-1H-pyridin-3-yl)benzimidazol-1-yl]methyl]piperidine-1-carboxylate (500 mg, 1.18 mmol, 1 eq) in HCl/dioxane (4 mol/L, 10 mL) . The mixture was stirred at 20 °C for 2 hr. LC-MS showed tert-butyl (3S)-3-[[2-(5-methyl-6-oxo-1H-pyridin-3-yl)benzimidazol-1-yl]methyl]piperidine-1-carboxylate was consumed completely. The reaction mixture was concentrated under reduced pressure to remove

HCl/dioxane. Compound 3-methyl-5-[1-[[(3R)-3-piperidyl]methyl]benzimidazol-2-yl]-1H-pyridin-2-one (460 mg, crude, HCl salt) was obtained as a white solid. $(M+H)^+$: 323.2.

(d) To a solution of 3-methyl-5-[1-[[(3R)-3-piperidyl]methyl]benzimidazol-2-yl]-1H-pyridin-2-one (500 mg, 1.39 mmol, 1 eq, HCl salt) and 1-isopropylpiperidine-4-carboxylic acid (238.58 mg, 1.39 mmol, 1 eq) in DMF (3 mL) was added HOBt (564.80 mg, 4.18 mmol, 3 eq) and EDCI (400.64 mg, 2.09 mmol, 1.5 eq) .The mixture was stirred at 20 °C for 6 hr. The reaction mixture was concentrated under reduced pressure to remove DMF. The residue was purified by prep-HPLC (column: Waters Xbridge Prep OBD C18 150*40mm*10um; mobile phase: [H_2O (10mM NH_4HCO_3)-ACN]; gradient:10%-40% B over 8.0 min). GSK023 pre [1-[[(3S)-1-(1-isopropylpiperidine-4-carbonyl)-3-piperidyl]methyl]benzimidazol-2-yl]-3-methyl-1H-pyridin-2-one (103 mg, 93.59% purity by LCMS) was obtained as a white solid. $(M+H)^+$: 476.3.

(e) To a solution GSK023 pre (95.2 mg, 0.2 mmol, 1 eq) in THF (2ml), add NaH (25.9 mg, 0.65 mmol, 3.2 eq) under an ice bath,stir at 0 °C for 15 min, then add CH3I (212ul, 0.34 mmol, 1.7 eq). The mixture was stirred at rt for 8 hr. Add MeOH TO quench concentration. The residue was purified by prep-HPLC (column: Waters Xbridge Prep OBD C18 150*40mm*10um; mobile phase: [H2O (10mM NH4HCO3)-ACN]; gradient:10%-40% over 8.0 min). GSK023 (S)-5-(1-((1-(1-Isopropylpiperidine-4-carbonyl)piperidin-3-yl)-methyl)-1H-benzo [d]imidazole-2-yl)-1,3-dimethylpyridin-2(1H)-one (54.9mg, 56.1% purity by LCMS) was obtained as a white solid. ^1H NMR (400 MHz, 393 K, DMSO-d_6): δ 7.98 (1H, d, J = 2.4 Hz), 7.61−7.68 (2H, m), 7.56−7.60 (1H, m), 7.20−7.29 (2H, m), 4.20−4.32 (2H, m), 3.92 (1H, br. d, J = 12.2 Hz), 3.58−3.67 (4H, m), 2.61−2.84 (5H, m), 2.13−2.23 (4H, m), 1.88−2.07 (3H, m), 1.39−1.63 (5H, m), 1.14−1.37 (3H, m), 1.09 (6H, d, J = 5.9 Hz). ^{13}C NMR (100.6 MHz, DMSO-d_6): δ additional peaks due to rotamers: 172.6, 172.0, 161.7, 150.4, 150.3, 142.4, 138.3, 136.7, 136.6, 135.8, 127.9, 127.8, 122.2, 121.9, 118.8, 111.2, 111.0, 108.1, 108.0, 62.0, 53.7, 47.9, 47.6, 47.5, 47.3, 46.8, 46.7, 45.1, 44.3, 41.4, 40.4, 40.3, 40.2, 40.1, 40.0, 38.1, 38.0, 37.8, 37.3, 35.8, 28.9, 28.8, 28.6, 28.0, 27.3, 25.4, 24.6, 24.2, 18.0, 17.8, 16.8. $(M+H)^+$: 491.0.

Compound ID: GSK023 pre

ET87616-2-P1B4 DMSO Bruker_J_400MHz T=273+80K

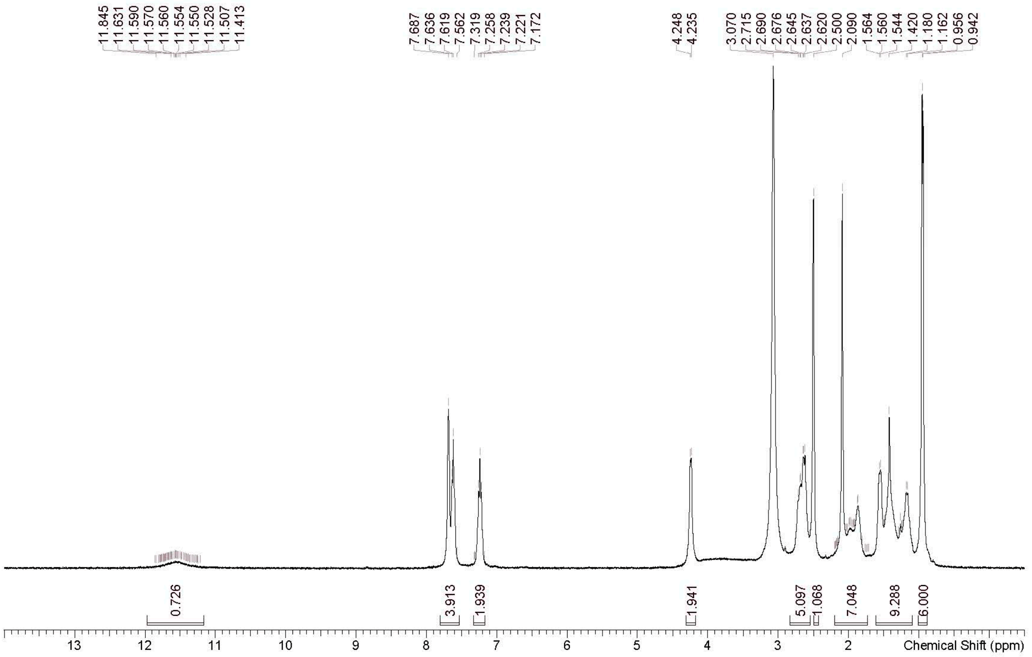

Acquisition Time (sec)	2.0972
Comment	ET87616-2-P1B4 DMSO Bruker_J_400MHz T=273+80K
Date	08 Dec 2023 09:19:12 (GMT+08:00)
Frequency (MHz)	400.2000
Nucleus	1H
Number of Transients	8
Origin	Avance
Original Points Count	16384
Owner	nmr
Points Count	65536
Pulse Sequence	zg30
Receiver Gain	101.00
SW(cyclical) (Hz)	7812.50
Solvent	DMSO-d6
Spectrum Offset (Hz)	2466.3508
Spectrum Type	standard
Sweep Width (Hz)	7812.38
Temperature (degree C)	80.043

LCMS REPORT

Compound ID	:	1
Sample ID	:	ET87616-2-P1A6
Injection Date	:	07. Dec. 2023
Location	:	P1-A-09
Inj. Vol.	:	1.00 uL
Acq Method	:	D:\Data\2301\231207 20\5_95AB_6MIN-220-254.M
Data Filename	:	D:\Data\2301\231207 20\ET87616-2-P1A6.D
Instrument	:	FL

==

Method Info : Instrument:Agilent 1260 HPLC MSD:6120 single quadrupole MSD
Column:Luna C18,2.0*50mm, 5µm
Column Temp: 40 °C
Mobile Phase:A:H_2O+TFA(0.04%)
Mobile Phase:B:ACN +TFA(0.02%)
Flow Rate:1.0ml/min

Time	B%	Flow(ml/min)
0.00	5	1.0
0.40	5	1.0
3.00	95	1.0
4.00	95	1.0
4.01	5	1.0
4.50	5	1.0

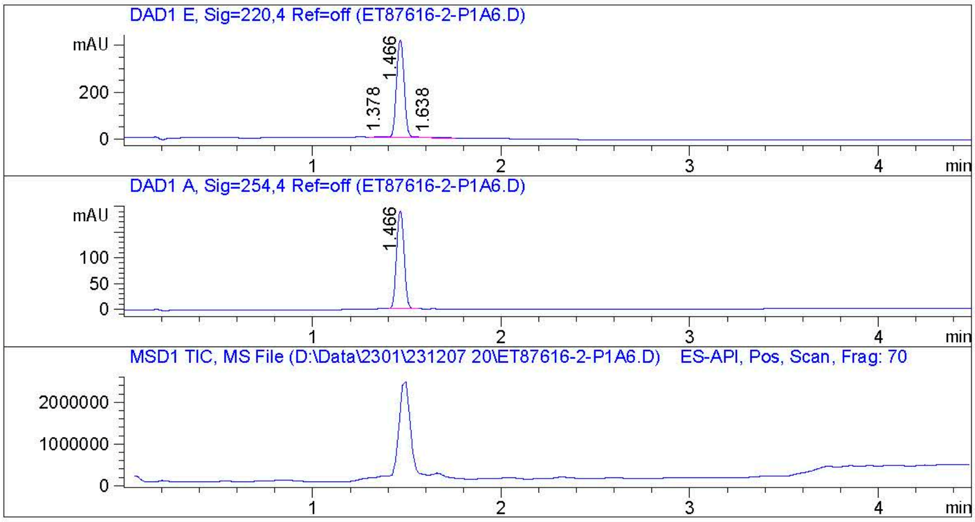

==

Report

==

Signal 1 : DAD1 E, Sig=220,4 Ref=off

Peak #	RT [min]	Height	Height %	Width [min]	Area	Area %
1	1.378	4.341	1.019	0.047	13.875	1.165
2	1.466	419.392	98.444	0.044	1167.993	98.059
3	1.638	2.289	0.537	0.059	9.241	0.776

Signal 2 : DAD1 A, Sig=254,4 Ref=off

Peak #	RT [min]	Height	Height %	Width [min]	Area	Area %
1	1.466	191.626	100.000	0.044	524.919	100.000

Signal 3 : MSD1 TIC, MS File

Peak #	RT [min]	Height	Height %	Width [min]	Area	Area %

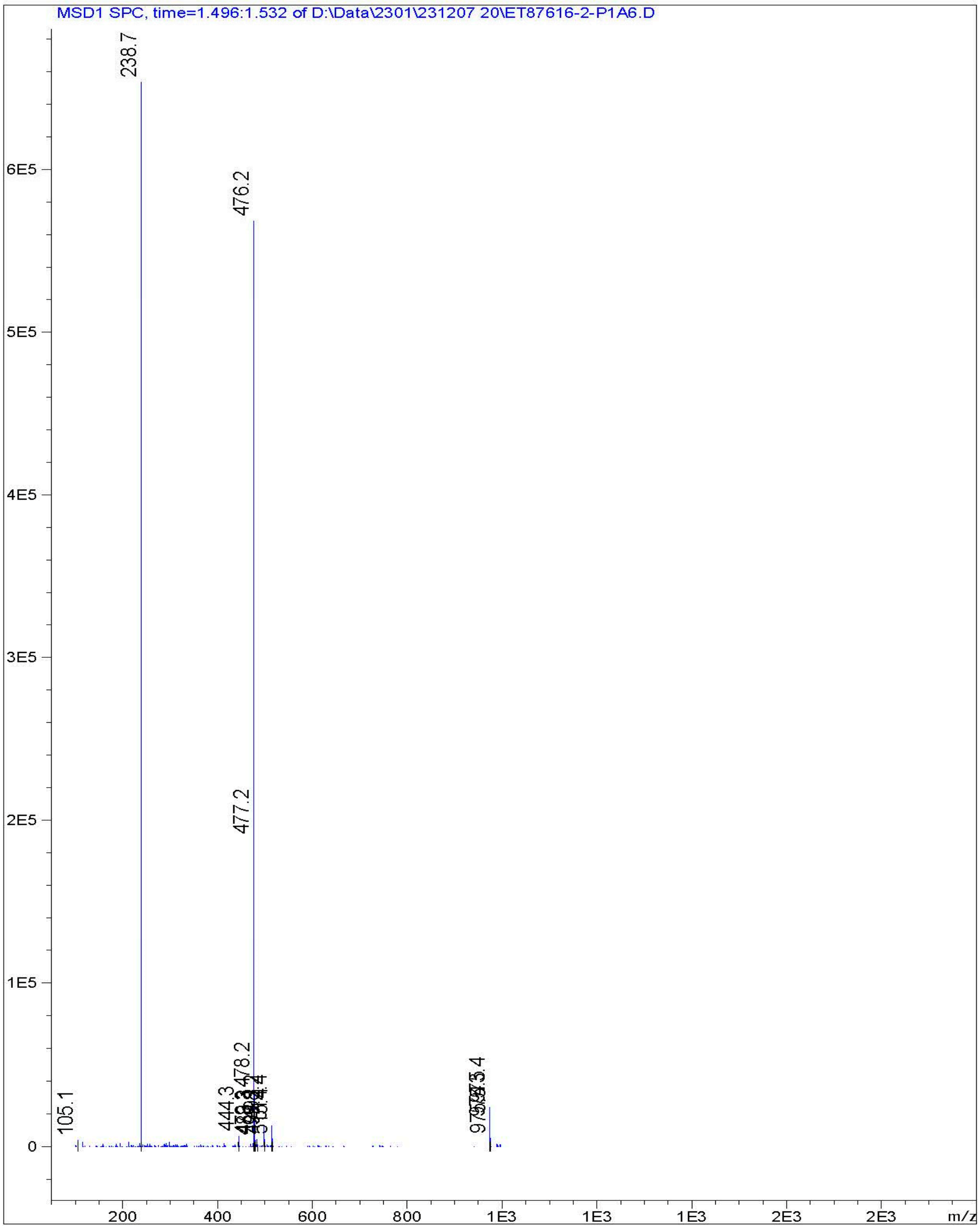

Supplement: Supplementary file 1 [file pharmaceuticals-17-01289-s001.zip › pharmaceuticals-3190613-supplementary.pdf]
